# Lysionotin Induces Ferroptosis to Suppress Development of Colorectal Cancer via Promoting Nrf2 Degradation

**DOI:** 10.1155/2022/1366957

**Published:** 2022-08-10

**Authors:** Zhuo Gao, Junnan Jiang, Lijian Hou, Fujian Ji

**Affiliations:** ^1^Department of Gastroenterology and Endoscopy Center, China-Japan Union Hospital of Jilin University, Changchun 130033, Jilin Province, China; ^2^Department of Gastrointestinal Colorectal Surgery, China-Japan Union Hospital of Jilin University, Changchun, 130033 Jilin Province, China; ^3^Department of Pathology, China-Japan Union Hospital of Jilin University, Changchun, 130033 Jilin Province, China

## Abstract

Extensive use of substances derived from natural sources has been documented in the treatment of colorectal cancer (CRC). Lysionotin (Lys) is a flavonoid present in the flowers and leaves of Gesneriaceae family plants. Despite its various pharmacological properties, which include neuroprotective, pro, antimalarial, and anticancer effects, the therapeutic advantages of Lys for CRC remain uncertain. In this present study, we demonstrated that Lys treatment successfully inhibited cell proliferation, migration, and invasion in HCT116 and SW480 CRC cells *in vitro*. Intriguingly, significant ferroptosis and reactive oxygen species (ROS) accumulation in CRC cells were induced by Lys treatment, whereas antagonism of ferroptosis by Liproxstatin-1 (Lip1) pretreatment retarded the anti-CRC effects of Lys. In addition, Lys reduced the amount of Nrf2 protein in CRC cells by increasing the rate at which it is degraded. Overexpression of Nrf2 rescued Lys reduced ferroptosis, suggesting the Nrf2 signaling is a crucial determinant of whether Lys induces ferroptosis in CRC cells. We also revealed that Lys suppressed tumor growth *in vivo* without obvious adverse effects on the main organs of mice. In conclusion, our results discovered that Lys treatment induced ferroptosis to exert antitumor effects in HCT116 and SW480 CRC cells by modulating Nrf2 signaling, providing a potential therapeutic approach for the prevention of colorectal cancer.

## 1. Introduction

In the year 2021, it was estimated that colorectal cancer (CRC) causes 52,980 deaths and is responsible for 11 percent of all new cancer diagnoses [1]. As a result, colorectal cancer is one of the kinds of cancerous tumors that has the highest mortality rate across the whole globe [2]. Adenocarcinomas, signet ring cell carcinomas, squamous cell carcinomas, and a few other less frequent types of colorectal cancer may be distinguished from one another by their histological features. Colorectal cancer is sometimes referred to by its medical abbreviation, CRC. Adenocarcinoma accounts for more than 90 percent of all CRC diagnoses, and many individuals are found to have advanced disease at the time of diagnosis [3]. In recent years, targeted therapy and immunotherapy have made significant strides towards becoming viable therapeutic options for CRC, complementing more traditional therapies such as chemotherapy, radiation therapy, and surgery [4]. Due to treatment resistance and evident systemic side effects, only 63 percent of CRC patients will live for five years or more after diagnosis. Consequently, there is an urgent need for innovative medicines that might prevent the course of the disease and save lives.

A rising amount of evidence demonstrates that phytochemical substances produced from natural sources are very efficient against cancer and other disorders [5–13]. Paclitaxel, a diterpenoid discovered in the bark of *Taxus brevifolia* in 1971, is the most prominent example of a drug that has been commercially successful [14]. It is often used in the treatment of malignant tumors such as cancer of the lung, esophagus cancer, breast cancer, and cancer of the pancreas. Produced by the soil fungus *Streptomyces peucetius*, doxorubicin is often used to treat a broad variety of solid cancers [15]. Camptothecin, podophyllotoxin, anhydrovinblastine, and vinorelbine are well-known anticancer medications derived from natural products [16]. Contrarily, there is evidence that colorectal cancer is resistant to a variety of chemotherapies [17]. Rising research has shown that phytochemicals including curcumin, resveratrol, bixin, and Ginkgo biloba offer great therapeutic potential for patients with CRC. The research and development of innovative drugs derived from natural chemicals is still required [16].

In contrast to apoptosis, necrosis, and autophagy, iron death is a unique kind of controlled cell death [18, 19]. The process is called ferroptosis because it is dependent on ferric ions. Dr. Brent R. Stockwell of Columbia University presented this theory for the first time in 2012 [20]. Iron death is caused by several factors, including glutathione depletion, decreased glutathione peroxidase (GPx4) activity, and the inability of lipid oxides to be metabolized by the GPx4-catalyzed glutathione reductase reaction [21]. This is then followed by iron oxidizing lipids via the Fenton reaction, which generates reactive oxygen species (ROS). Iron toxicity is the outcome of having an excessive amount of iron in the body, and circulating iron binds to transferrin as Fe^3+^, and excess iron is deposited in ferritin without taking part in ROS generation activities through transferrin receptor 1 [22]. Intriguingly, ferroptosis is also deeply involved in p53-mediated tumor suppression [23, 24]. Due to its important role in cell fate determination, ferroptosis is expected to be a valuable drug target in the treatment of cancers [21].

Mounting studies demonstrated that natural compounds targeting ferroptosis can be used for treatment of solid tumors such as lung cancer [25], bladder cancer [26], and pancreatic cancer [27]. However, evidence regarding the anti-CRC activities of ferroptosis-targeted natural compounds is still deficient. In this study, we reported lysionotin (Lys), a flavonoid isolated from *Lysionotus pauciflorus* Maxim (Gesneriaceae), exerts tumor-suppressing characteristics in CRC cell line HCT116 and SW480 cells. Intriguingly, Lys treatment induced potent ferroptosis in CRC cells, and inhibition of ferroptosis retarded the anti-CRC activities *in vitro* and *in vivo*. We further demonstrated that Lys-induced ferroptosis is dependent on the promotion of nuclear factor erythroid 2–related factor 2 (Nrf2) degradation. These results suggest that Lys induced ferroptosis to suppress the progression of CRC via regulating Nrf2 signaling, which paves the way for a new potential strategy for CRC therapy.

## 2. Materials and Methods

### 2.1. Regents

Selleck Chemicals Co., Ltd. (TX, USA) provided MG132, Lys (>97% pure), and Liproxstatin-1 (Lip1). For treating cells, Lip1 and MG132 were diluted using dimethyl sulfoxide (DMSO). Lys and Lip1 were diluted in dilution buffer (5 percent DMSO, 40 percent PEG 300, 5 percent Tween 80, and 50 percent H_2_O) for animal studies. Abcam (Cambridge, United Kingdom) provided antibodies for cystine/glutamate transporter (xCT, catalog number: ab175186), glutaminase (catalog number: ab93424), and glutathione peroxidase 4 (GPX4, catalog number: ab125066). Cell Signaling Technology (CST, Danvers, United States) provided antibodies for Ubiquitin (Ub, catalog number 3936S), Ferritin Heavy Chain 1 (FTH1, catalog number 4393S), Nrf2 (catalog number 12721), and glyceraldehyde 3-phosphate dehydrogenase (GAPDH, catalog number 5174S).

### 2.2. Cell Cultures

We obtained human CRC HCT116 and SW480 cell lines, as well as HIEC and NCM460 cell lines from normal human colon epithelial tissue, from the American Type Culture Collection (ATCC). Cells were cultured in DMEM with 10 percent fetal bovine serum (Sigma-Aldrich, St. Louis, MO, USA) at a temperature of 37 degrees Celsius in an environment containing 5 percent carbon dioxide.

### 2.3. Assay of Cell Viability

To determine the toxicity of Lys in CRC cells, a CCK-8 kit purchased from Sigma-Aldrich was used. In an effort to maintain clarity, each well of the 96-well plates was populated with 2,000 cells. Following a Lys-containing or Lys-free incubation period of 24 hours, CCK-8 reagent was added to each well, and the cells were allowed to continue growing at 37 degrees Celsius for an additional 2 hours. In order to determine the 450 nm absorbance of each well, a microplate reader (BioTek, Winooski, VT, USA) was used.

### 2.4. Colony Formation Assay

Colony formation assay was performed in HCT116 and SW480 cells according to a previous report [28]. Briefly, cells were seeded into 6-well plates (400 cells each well). Immediately upon seeing the formation of colonies on the Petri plate, the culture was terminated. PBS was used to wash the cells for a total of 3 times. After that, the cells were fixed for a further 15 minutes using a 1 : 3 solution of acetic acid and methanol. To stain the cells, the fixation solution was removed, and a staining solution containing 0.1 percent crystal violet was applied to them at room temperature for a period of 30 minutes. Following the final wash with PBS, an inverted microscope (Olympus, Tokyo, Japan) was used to achieve the cell images, and the number of colonies that had more than 60 cells in each well was counted.

### 2.5. Measurement of Ferrous Ion

FerroOrange, manufactured in Japan by Dojindo, was applied to cells in order to ascertain the quantity of cellular iron. Briefly, cells were subjected to a treatment with 1 mM of FerroOrange in serum-free media for a period of 30 minutes at 37 degrees Celsius. A fluorescence microscope (Olympus) was used for the detection of fluorescence of the CRC cells.

### 2.6. Transduction

The gene coding Nrf2 was inserted into pAPH vectors [29] for overexpressing Nrf2 in CRC cells. We temporarily transfected an empty vector or a vector that expresses Nrf2 into HCT116 and SW480 cells using Lip3000 (Invitrogen) as directed by the company's protocol.

### 2.7. Wound Healing Analysis

Wound healing analysis was performed in HCT116 and SW480 cells according to a previous study [30]. Briefly, either HCT116 or SW480 cells was cultured in 6-well plates until they achieved a confluency level of about 90 percent. After that, a p200 pipette tip was used to scrape the cell monolayer in a linear fashion. Before placing the cells back into the incubator at 37 degrees Celsius for a further 24 hours, the cell debris was removed with PBS. Immediate photographs of the damage were taken using a microscope (Olympus).

### 2.8. Quantitative Polymerase Chain Reaction (qPCR)

Using the RNA isolation solution TRIzol (Invitrogen, USA), total RNA was extracted from cells or homogenized tissue samples, and then, it was used as a template for the synthesis of cDNA using the MonScriptTM RTIII All-in-One Mix with dsDNase (Monad biotech, China). qRT-PCR was carried out with the MonAmpTMFast SYBR® Green qPCR Mix (manufactured by Monad biotech) on a CFX connect system (Bio-Rad, USA). The primer sequence information is listed in Table 1. The relative gene expression was calculated using the 2^-*ΔΔ*Ct^ method.

### 2.9. Transwell Assay

*In vitro* cell migration and invasion were investigated via the use of a Transwell experiment, as was mentioned before [31]. 2 × 10^4^ cells in DMEM devoid of serum were plated into the top chamber of Corning Transwell Inserts. After that, the top chambers were placed on a 24-well plate, and the bottom chamber was filled with DMEM containing 10 percent FBS. After spending 24 hours in an incubator set to 37 degrees Celsius, the membrane was removed and colored using a crystal violet solution. The images were captured with an Olympus microscope. The number of migratory cells was determined by counting the cells in six random areas. The migration test and the invasion test were both carried out in the same manner, with the exception that Matrigel was utilized to cover the membrane of the top chamber.

### 2.10. Measurement of Glutathione (GSH) and Malondialdehyde (MDA) Levels

Cells with or without treatment were lysed using the RIPA buffer (CST). The BCA assay kit (Beyotime Biotechnology, Shanghai, China) was used to measure the amount of total protein. The intracellular GSH and MDA levels were determined using a GSH assay kit and an MDA kit (Beyotime), respectively, according to the manufacturer's instructions.

### 2.11. Western Blot

The Western blot analysis was performed as described before [32, 33]. Briefly, after boiling the samples in 5× loading buffer, an appropriate quantity of each sample was loaded onto a discontinuous sodium dodecylsulfate-polyacrylamide gel (SDS-PAGE), and the proteins were transferred to PVDF membranes (Millipore, Bedford, MA, USA). It took one hour of using nonfat dry milk dissolved in PBS-Tween-20 (PBST) at a concentration of 5% for the membrane blocking procedure. After this, three washes with PBST were performed, and then either primary (1 : 2000) or monoclonal (1 : 5000) anti-GAPDH antibodies were incubated overnight at 4 degrees Celsius with the samples. Following washing with PBST, the membranes were incubated at room temperature with the secondary antibody that was coupled to horseradish peroxidase for a period of two hours (1 : 5000). With the assistance of the BioRad ChemiDoc MP Image System, the chemical signals were at long last discovered.

### 2.12. Determination of Reactive Oxygen Species (ROS) Generation

With the use of the fluorescent probe 2′, 7′-dichlorofluorescin diacetate (DCFH, Beyotime), levels of ROS were determined in CRC cells as previously described [34]. At a temperature of 37 degrees Celsius, the cells were treated with 5 M DCFH for an hour. After washes with PBS, cells were observed under a fluorescence microscope and the cell images were taken for quantitative analysis of the fluorescence using ImageJ.

### 2.13. Xenograft Mouse Models

Charles River Laboratories provided male athymic nude mice that were four weeks old weighing 18-22 g and housed in animal facilities at temperatures between 20 and 25 degrees Celsius, with humidity between 50 and 60 percent and a light/dark cycle of 12 hours on and 12 hours off. The mice had unrestricted access to clean food and water, and the Animal Care and Use Committee of the China-Japan Union Hospital of Jilin University granted approval for the animal trials (Changchun, China). Under the skin of each mouse's left flank, 0.2 milliliters of HCT116 cells suspended in PBS and Matrigel were injected. The cells were kept alive by suspending them in PBS containing Matrigel. At 6 days post the xenograft, the mice received intraperitoneal (i.p.) injections of Lys (20 mg/kg) with or without Lip1 (10 mg/kg) every three days for five times. The size of the tumor was measured using a Vernier caliper every three days. The following formula was used to calculate the size of the tumor: tumor volume (mm^3^) = maximum length (mm) × perpendicular width (mm) 2/2. On day 24, after the administration of the injection, the tumors were surgically opened up and photographs were obtained of their interiors.

### 2.14. Hematoxylin and Eosin (HE) Staining

Histology analysis was used in a standard way to measure organ damage. In short, hematoxylin was put on the piece of tissue, which was then put in an incubator for 5 minutes. The slides were then cleaned twice with water that had been distilled. After that, a bluing reagent was put on the tissue slice for 10 seconds. After being washed, the slides were left in eosin Y reagent for 2 minutes. The slides were then put in pure alcohol to dry them out. After sealed with resin, the slides were photographed with a microscope.

### 2.15. Statistical Analysis

GraphPad Prism 8, a tool developed by GraphPad Software, was used for statistical analysis, and all of the data were presented as means and standard deviations (SD). When looking at the data, the one-way analysis of variance (ANOVA) test with post hoc multiple comparisons was employed, and the Student *t*-test was used when comparing the two groups. It was deemed statistically significant to use a *P* value of 0.05, thus, that is what was utilized.

## 3. Results

### 3.1. Lys Restrained the Proliferation of HCT116 and SW480 Cells *In Vitro*

**Figure 1:**
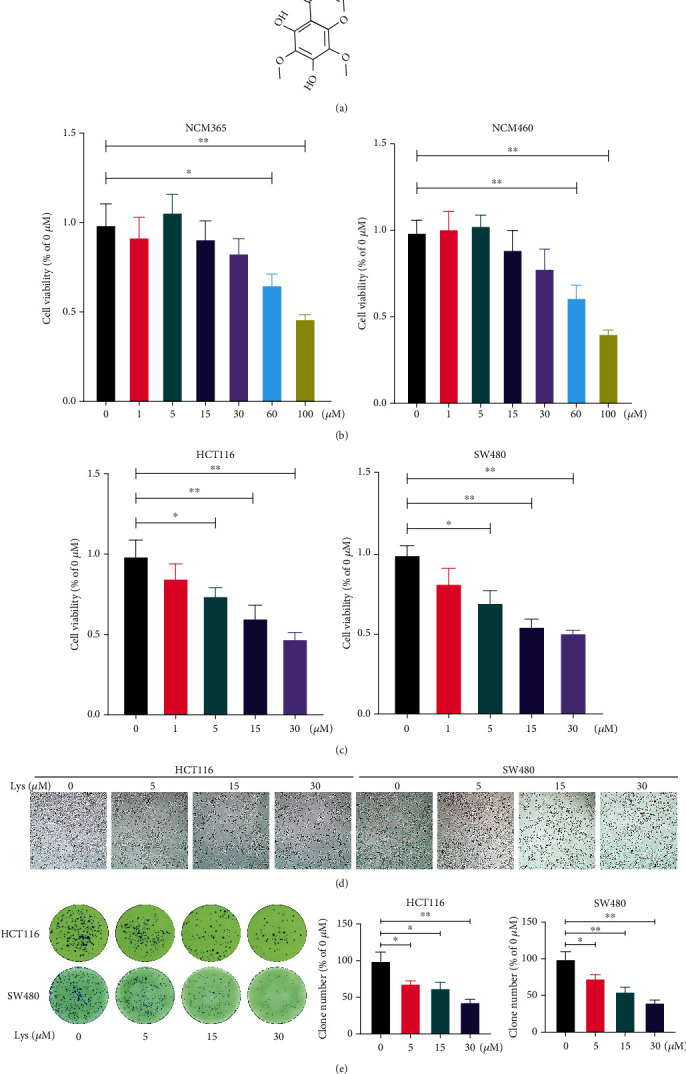
Lys inhibits cell viability in HCT116 and SW480 cells. (a) Chemistry formula of Lys. After treatment with the relevant dosages of Lys *in vitro* for a period of 24 hours, the CCK-8 assay was used to determine the level of cell viability of HIEC and NCM460 cells (b), as well as HCT116 and SW480 Cells (c). (d) A microscope was used to evaluate the morphology of the cells. (e) Crystal violet staining was used in order to investigate the cell colony development. *P* values: ^∗^*P* ≤ 0.05 and ^∗∗^*P* ≤ 0.01.

The chemistry formula of Lys is shown in Figure 1(a). The CCK-8 assay was used to measure the cytotoxicity of Lys in normal colon epithelial cells and CRC cell line cells. The results revealed that the cell viability was not decreased in the HIEC and NCM460 cells that were treated with Lys at dosages that were lower than 60 *μ*M. (Figure 1(b)). However, 5 *μ*M, 15 *μ*M, or 30 *μ*M concentrations of Lys effectively inhibited the proliferation of HCT116 and SW480 cell lines *in vitro* (Figure 1(c)). In line with this finding, morphological analysis showed that CRC cells were sensitive to Lys treatment at a dosage more than 5 *μ*M (Figure 1(d)). Furthermore, Lys significantly decreased the growth of HCT116 and SW480 cell colonies by a lot (Figure 1(e)). So, our results showed that Lys therapy effectively stopped CRC cells from growing *in vitro*.

### 3.2. Lys Treatment Suppresses the Motility of HCT116 and SW480 Cells

**Figure 2:**
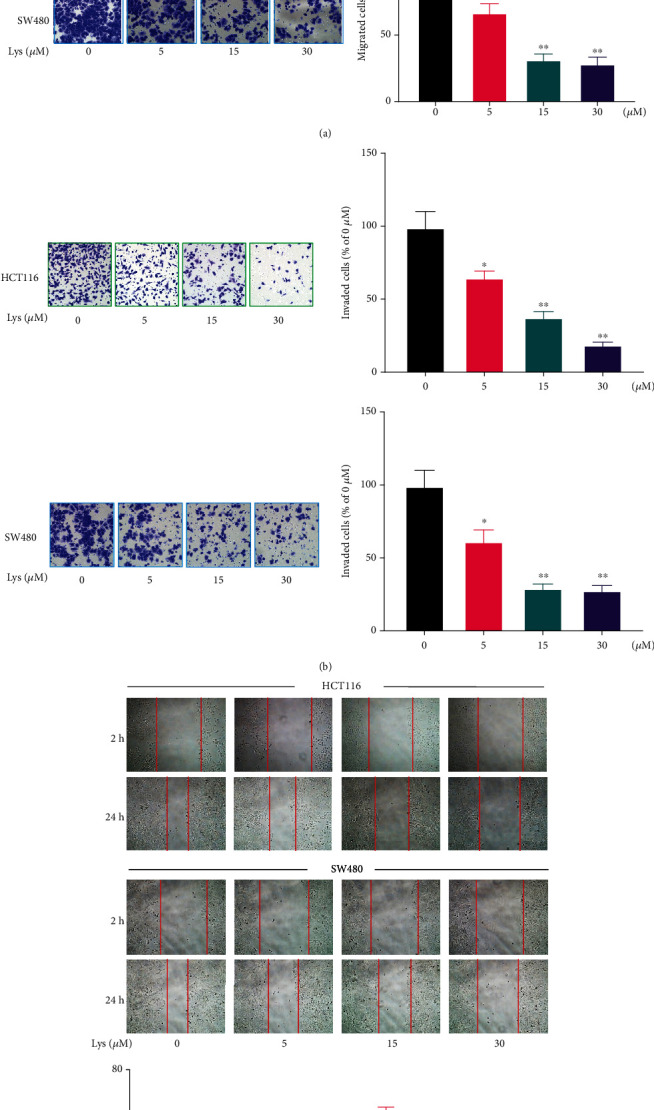
Lys treatment suppresses the migration and invasion of CRC cells. At 24 hours after incubating HCT116 and SW480 cells with Lys at concentrations ranging from 0-30 *μ*M, wound healing analysis (a) was used to analyze the effects of a therapy called Lys on the migration of CRC cells. The use of Transwell assays allowed for the investigation of the effects of Lys on the migration (b) and invasion (c) of CRC cells. *P* values: ^∗^*P* ≤ 0.05 and ^∗∗^*P* ≤ 0.01.

Using wound healing, Transwell migration, and invasion experiments with HCT116 and SW480 cells, the effects of Lys treatment on CRC cells motility were investigated. When HCT116 and SW480 cells were treated with Lys, the wounds that were caused by scratches took much longer to heal in comparison to when the cells were treated with a vehicle (Figure 2(a)). A dose-dependent reduction in the number of migrating (Figure 2(b)) and invading (Figure 2(c)) cells was seen in response to treatment with Lys, as shown by the findings of the Transwell test (see above). According to the findings, Lys significantly reduced the ability of CRC cells to migrate and disseminate *in vitro*.

### 3.3. Lys Induced Ferroptosis in CRC Cells

**Figure 3:**
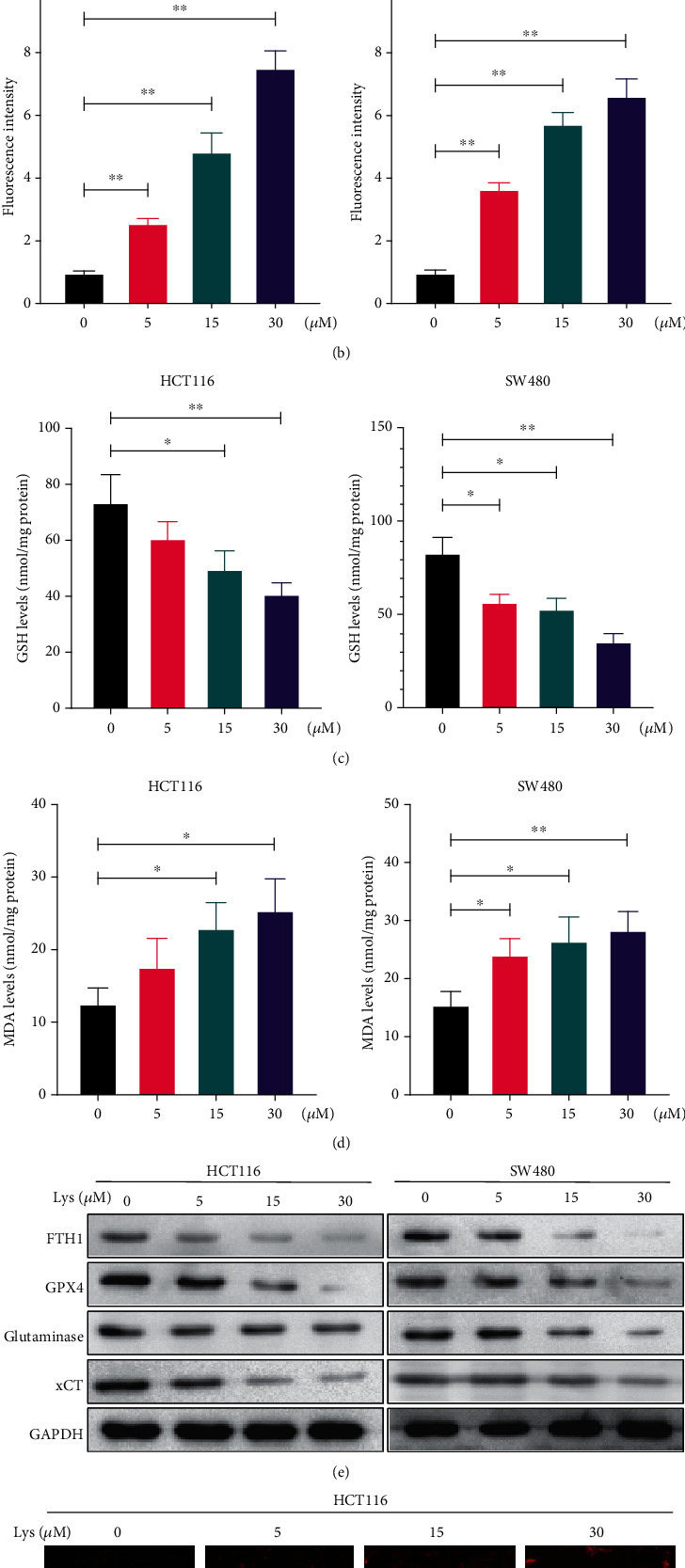
Lys triggered ferroptosis in CRC cells. (a, b) ROS levels were measured by DCFH-DA staining after HCT116 and SW480 cells had been treated with Lys for 24 hours. The results are shown as the mean standard deviation. (c) The GSH level in HCT116 and SW480 cells was determined after the treatment with Lys for 24 hours, and the results showed that there was a significant difference between the groups. (d) The MDA level in HCT116 and SW480 cells was measured following the treatment with Lys for 24 hours. (e) Following a treatment with Lys for 24 hours, the iron content of HCT116 and SW480 cells was evaluated using the ferroOrange staining method. (f) Western blotting was used in order to identify a number of proteins connected to ferroptosis. ^∗^*P* < 0.05 and ^∗∗^*P* < 0.01.

In ferroptosis, it is well known that lipid peroxidation and GSH depletion are important processes. After Lys was added to HCT116 and SW480 cells, the amount of ROS, GSH, and MDA inside the cells was measured. The results showed that after treatment with Lys, ROS went up (Figures 3(a) and 3(b)) and GSH went down (Figure 3(c)). Lys treatment, on the other hand, clearly raised the intracellular MDA levels (Figure 3(d)). Western blotting was used to determine the effects of Lys on ferroptosis makers. As shown in Figure 3(e), after treatment with Lys, the protein levels of FTH1, GPX4, glutaminase, and solute carrier family 7 member 11 (xCT/SLC7A11) in HCT116 and SW480 cells were significantly lower than in the control group. Ferroptosis is a buildup of cells that depends on iron. So, an iron probe, FerroOrange was used to measure the amount of iron inside HCT116 and SW480 cells. Lys-treated cells gave off a lot more orange light than cells that had not been treated (Figure 3(f)). In conclusion, the results of our studies demonstrated that Lys induced CRC cells to transition towards ferroptosis.

### 3.4. Inhibition of Ferroptosis Abrogates the Anti-CRC Effects of Lys *In Vitro*

**Figure 4:**
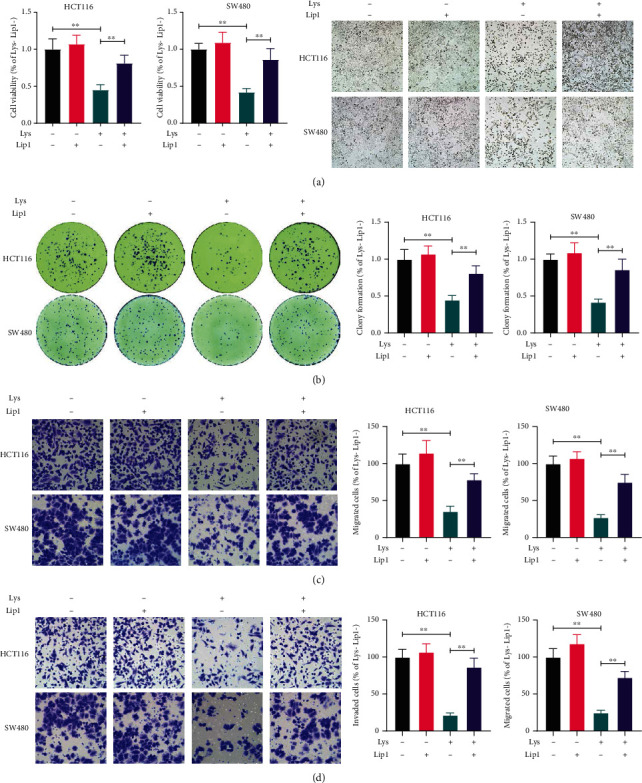
Lip1 pretreatment abolished Lys-mediated suppression of CRC cells. Prior to the addition of 30 M of Lys, HCT116 and SW480 cells were pretreated with Lip1 at a concentration of 200 nM for six hours. The CCK-8 test and the clonogenic assay were used to determine the level of cell viability (b) and survival (c), respectively, 24 hours after Lys was administered. The Transwell test was used to ascertain the level of motility present in CRC cells (c, d). The data are either pictures that are typical of the whole or are given as the mean plus the standard deviation. ^∗^*P* < 0.05, ^∗∗^*P* < 0.01.

To confirm the involvement of ferroptosis in the inhibitory effects of Lys in CRC, we pretreated the cells with Lip1, a ferroptosis inhibitor, before administering Lys. Lip1 pretreatment, as demonstrated in Figures 4(a) and 4(b), reversed the Lys-induced deficiency in proliferation and clonogenic ability of HCT116 and SW480 cells *in vitro*. Furthermore, Lip1 pretreatment reduced Lys' inhibitory effects on CRC cell migration (Figure 4(c)) and invasion (Figure 4(d)).

### 3.5. Lys Reduces Nrf2 Protein Levels by Promoting Nrf2 Protein Degradation

**Figure 5:**
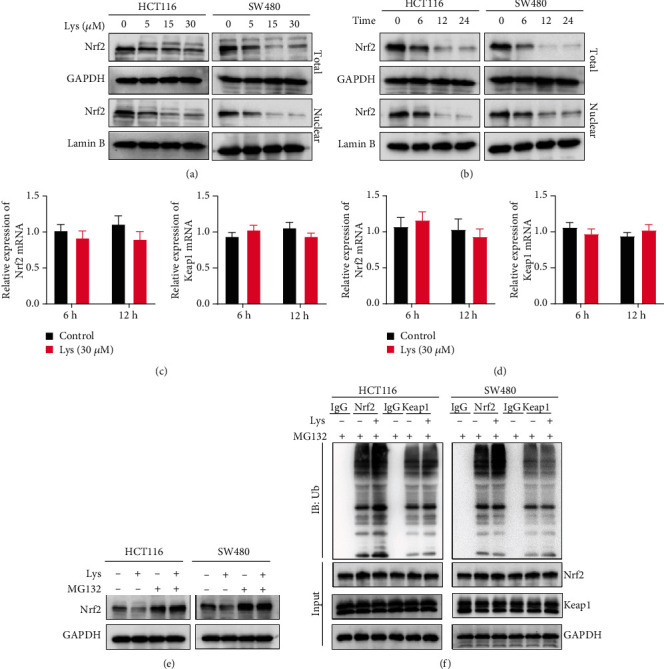
Lys inhibits Nrf2 protein levels in HCT116 and SW480 cells. (a) HCT116 and SW480 cells were treated with varying doses of Lys (ranging from 0 to 30 *μ*M) over a period of 24 hours, and western blot analysis was done to determine the amount of the Nrf2 protein produced. (b) HCT116 and SW480 cells were treated with 30 *μ*M of Lys for 6 hours, 12 hours, and 24 hours, and western blot analysis was done to determine how much Nrf2 protein was produced. qRT-PCR was performed to quantify the mRNA levels of Nrf2 and Keap1 in CRC cells after HCT116 and SW480 cells were treated with 30 *μ*M of Lys for a duration of 6 hours and 12 hours (c, d). (e) HCT116 and SW480 cells were treated with 30 *μ*M of Lys, 10 *μ*M of MG132, or a combination of Lys and MG132 for a period of 12 hours, and then, the protein levels of Nrf2 were analyzed by western blotting. (f) The ubiquitination test was used to determine the levels of ubiquitination of Nrf2 in HCT116 and SW480 cells after they were treated with or without 30 *μ*M of Lys for a period of 12 hours. The data are presented as the mean standard deviation, with a sample size of three.

Nrf2 is a key regulator in cell redox and ferroptosis. The effects of Lys on the expression of the Nrf2 protein were then seen in HCT116 and SW480 cells. As shown in Figure 5(a), at concentrations of 5, 15, and 30 *μ*M, Lys drastically downregulated the amount of Nrf2 protein in cells. Also, the amount of Nrf2 protein in cells was much lower at 6, 12, and 24 h after being treated with 30 *μ*M Lys (Figure 5(b)). Additionally, quantitative reverse transcription-PCR was used to determine the mRNA levels of Nrf2 and Keap1 in Lys-treated HCT116 and SW480 cell cultures. Because the findings revealed that there was no significant difference between the mRNA levels of Nrf2 and Keap1 in Lys-treated cells and control cells (Figures 5(c) and 5(d)), indicating that Lys does not inhibit the transcription of Nrf2. In light of this, we investigated whether or not Lys decreases the quantity of Nrf2 protein by affecting its protein stability. As shown in Figure 5(e), the proteasomal inhibitor MG132 protected the levels of Nrf2 in CRC cells from Lys treatment. Also, research on ubiquitination showed that the levels of ubiquitination of Nrf2 in cells were much higher after Lys treatment (Figure 5(f)). Also, when cells were treated with Lys, the level of ubiquitination of Keap1 did not change much (Figure 5(f)). These results suggest that Lys downregulated the amount of Nrf2 protein in CRC cells by speeding up the degradation of Nrf2 protein.

### 3.6. Overexpression of Nrf2 Retarded Lys Induced Ferroptosis

**Figure 6:**
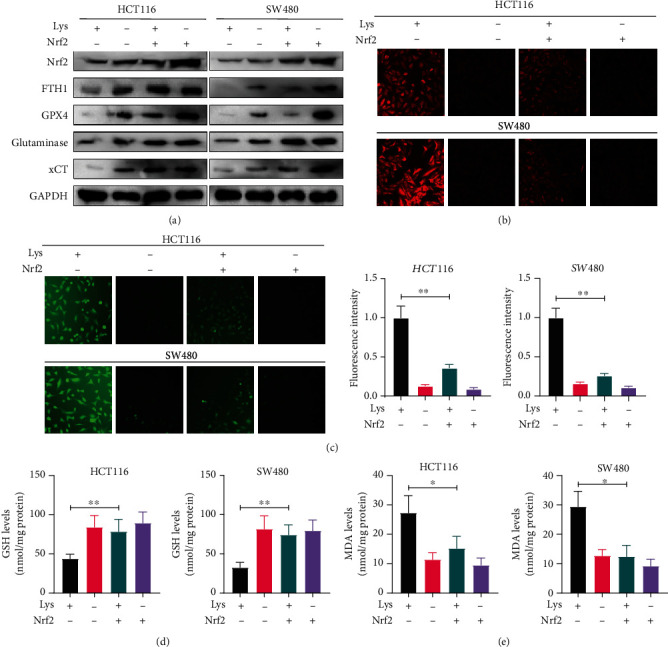
Lys triggered ferroptosis via the Nrf2 pathway. After transfecting the HCT116 and SW480 cells with an empty vector or Nrf2 for 18 hours, the cells were treated with Lys for an additional 24 hours. (a) Using immunoblotting, we examined the levels of Nrf2, GPX4, ferritin, and glutaminase expression in HCT116 and SW480 cells. (b) FerroOrange staining was used to determine the amount of iron present in HCT116 and SW480 cells. (c) A flow cytometer was used to determine the ROS level; the mean and standard deviation are shown here, ^∗^*P* < 0.05. (d, e) Commercial kits were used to determine the GSH levels and MDA levels in HCT116 and SW480 cells, respectively. After being treated with Lys for 24 hours, the results were as follows: ^∗^*P* < 0.05, ^∗∗^*P* < 0.01.

Recent studies have demonstrated that inhibiting the activity of the Nrf2 signaling pathway is essential to preventing cancer from expanding and halting the process of ferroptosis. We employed a Nrf2 expressing vector to specifically boost the activation of Nrf2 in order to get a better understanding of the relationship that exists between the Lys-induced ferroptosis and the Nrf2 signaling. We discovered that increasing the amount of Nrf2 in the cell led to an increase in the expression of GPX4, ferritin, xCT, and glutaminase, while Lys treatment made the expression of all of these genes go down (Figure 6(a)). By overexpressing Nrf2, both Lys-induced increases in intracellular iron (Figure 6(b)) and ROS (Figure 6(c)) were reversed. Also, Nrf2 overexpression helped a lot to fix the lower GSH levels and higher MDA levels that Lys treatment caused (Figures 6(d) and 6(e)). Based on these results, it seems likely that Lys-induced ferroptosis in CRC cells is mostly controlled by modulating Nrf2 signals.

### 3.7. Lys Inhibits Tumor Growth *In Vivo* by Activating Ferroptosis

**Figure 7:**
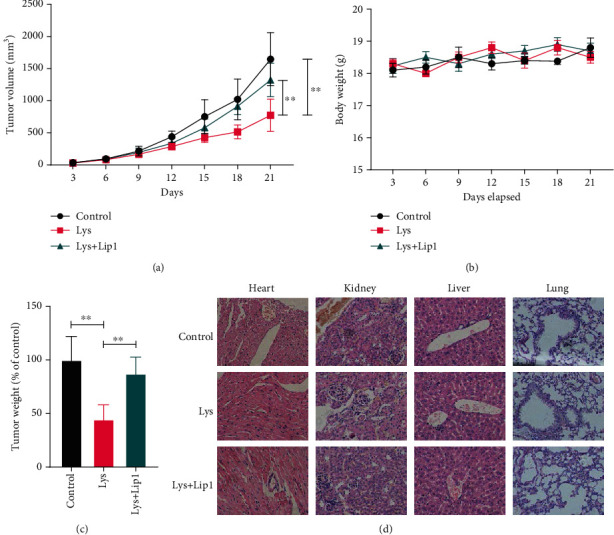
Lys suppresses CRC development *in vivo* through priming ferroptosis. Seven days following the xenograft, the patient received an intraperitoneal (i.p.) injection of Lys containing 20 mg/kg once every three days for a total of four doses. In order to suppress ferroptosis, Lip1 (10 milligrams per kilogram) was injected intraperitoneally (i.p.) once day after the administration of Lys up to the time of sacrifice. At the end of the 24th day following the xenograft, all of the mice were put to sleep, and the weight and volume of the tumor were measured. (c) The total number of treatments continued till the body weight of the mice was recorded. (d) Histological data of H&E staining performed on samples taken from various experimental groups' hearts, kidneys, and livers; *P* values: ^∗^*P* ≤ 0.05, ^∗∗^*P* ≤ 0.01.

The antitumor properties of Lys were studied further *in vivo*. The results showed that administration of Lys to mice with SW480 tumor xenografts greatly reduced tumor growth, whereas Lip1 treatment decreased Lys' antitumor potential (Figures 7(a) and 7(b)). The weight of the mice did not change when Lys and/or Lip1 were used (Figure 7(c)). Also, the results of histopathology studies showed that treatment with Lys and/or Lip1 showed no obvious toxicities to the main organs of mice, including the lung, heart, liver, and kidney (Figure 7(e)), suggesting that Lys treatment with efficient dosage does not exert side effects *in vivo*.

## 4. Discussion

Therapeutic regents derived from phytomedicine have been used in China for thousands of years [35, 36]. The fact that Lys is a trimethoxyflavone denotes that it has methoxy groups at positions 6, 8, and 4′ in addition to hydroxy groups at positions 5 and 7 in the chemical structure [37]. It functions as a phytometabolite and consists of both trimethoxyflavone and dihydroxyflavone. Lys naturally exists as a free chemical or a glycoside in the leaves and flowers of the Gesneriaceae family [38]. Clearly, Lys suppresses *Staphylococcus aureus* [38], and the activity of cytochrome P450 enzymes *in vitro* [37]. Lys also mediated mitochondrial apoptosis in hepatocellular carcinoma cells via activating caspase-3 [39]. Despite these various effects, the therapeutic benefits of Lys for CRC remain undetermined. *In vitro* cell viability experiments utilizing cell cultures demonstrated that Lys treatment may inhibit the development and survival of CRC cells without harming normal colon epithelial cells. In contrast, wound healing, Transwell migration, and invasion experiments were performed on HCT116 and SW480 cells to determine the effects of Lys treatment on CRC cell movement and invasion. Results surface Lys-treated HCT116 and SW480 cells displayed a slower rate of wound healing than vehicle-treated cells. In addition, the Transwell test results revealed that Lys treatment dose dependently decreased the number of migrating and invading cells. We made the discovery that Lys has the potential to inhibit the formation of tumors and the invasion of CRC cells *in vivo*.

Recent research has revealed that triggering ferroptosis dramatically reduces the risk of developing a tumor, as well as increases the efficiency of both targeted treatment and chemotherapy [40]. It has been shown, for example, that vitamin C may trigger ferroptosis and that treating RAS/BRAF wild-type CRC patients with a combination therapy consisting of vitamin C and cetuximab may prevent the development of acquired resistance to cetuximab in such patients [41]. Upregulation of MT-1G expression results in a reduction in ferroptosis, which in turn protects hepatocellular carcinoma cells from the action of sorafenib and contributes to the progression of cancer [42]. The use of erastin, even for a short period of time, amplifies the cytotoxic effects of cisplatin to a significant degree [43]. Ferroptosis is induced in p53 mutant hypopharyngeal squamous cell carcinoma cells by treatment with paclitaxel and RSL3 at low doses [44]. When compared to the control group, the Lys treatment led to a decrease in the amount of protein that was found in FTH1, GPX4, glutaminase, and solute carrier family 7 member 11 (xCT/SLC7A11) in HCT116 and SW480 cells. In addition, the iron probe FerroOrange was used to examine the quantities of intracellular iron present in HCT116 and SW480 cells. When compared to untreated cells, cells that had been treated with Lys produced a much higher amount of orange fluorescence. The inhibitory effects of Lys on the migration and invasion of CRC cells were reduced when the cells were pretreated with Lip1. This provides more evidence that ferroptosis occurred in CRC cells that had been treated with Lys.

External stimuli (e.g., drugs, UV, and ionizing radiation) and endogenous free radicals and reactive oxygen species (ROS) can directly or indirectly damage cellular components such as proteins, lipids, and DNA [45]. To defend against these adverse effects, the body has developed a complex set of oxidative stress response systems to mitigate cell damage. In addition, Nrf2, which is a crucial transcription factor in the regulation of antioxidant stress, plays a crucial role in the induction of the body's antioxidant response [46, 47]. This includes the regulation of redox balance, drug metabolism and excretion, energy metabolism, iron metabolism, amino acid metabolism, survival, proliferation, autophagy, proteasomal degradation, DNA repair, and mitochondrial physiology, among other processes [48]. In addition, the Keap1-Nrf2 system has recently emerged as an important therapeutic target in the treatment of a variety of diseases, including cancer, neurological disorders, autoimmune diseases, and inflammatory conditions [49]. Activation of the Keap1-Nrf2 pathway is one of the most essential antitumorigenesis pathways [49]. Not only synthetic drugs, such as oltipraz, but also plant-derived substances, such as sulforaphane, curcumin, resveratrol, etc., regulate genes and have chemopreventive effects by targeting the Nrf2 pathway [50]. Enhancing the activity of Nrf2 is a tried-and-true method for preventing chronic illnesses and cancer originating from oxidative and inflammatory stress [48]. However, constitutive activation of Nrf2 in diverse tumors will boost cancer cell proliferation and result in chemoresistance and radioresistance of cancer cells. In our work, we discovered that Lys reduces Nrf2 protein levels in CRC cells by promoting their degradation. Lys at doses of 5, 15, and 30 *μ*M significantly decreased cellular Nrf2 protein levels. At 6, 12, and 24 hours after a 30 *μ*M Lys treatment, the Nrf2 protein level in cells was seen to be significantly reduced. In addition, quantitative real-time polymerase chain reaction (qRT-PCR) was used to detect the mRNA levels of Nrf2 and Keap1 in HCT116 and SW480 cells after treatment with Lys. The results showed that there was no significant difference in the mRNA levels of Nrf2 and Keap1 between Lys treated cells and control cells, which suggests that Lys does not inhibit the transcription of Nrf2. Determining if activation of Nrf2 causes cancer, how to target Nrf2 (direct or indirect suppression of upstream protein kinases), and elucidating the structure of Keap1 have therefore been the focus of academic research. Following Lys therapy, Nrf2 ubiquitination levels were significantly raised, according to study on ubiquitination. Keap1 ubiquitination levels did not change significantly after Lys treatment, indicating that Keap1 is not required for Lys-induced degradation of the Nrf2 protein.

Nrf2 served as a key regulator in ferroptosis, due to its functions in maintaining the redox homeostasis and directly regulating the ferroptosis related genes such as GPX4, xCT, ferritin, and glutaminase [51, 52]. In order to get a deeper understanding of the connection between Lys-induced ferroptosis and Nrf2 signaling, we transfected a Nrf2 expressing vector into the cells in order to selectively stimulate Nrf2 activity. We found that Nrf2 overexpression boosted the expression of Nrf2, GPX4, ferritin, xCT, and glutaminase, which were all reduced by Lys treatment. The buildup of intracellular iron and reactive oxygen species produced by Lys was reduced by Nrf2 overexpression. In addition, Nrf2 overexpression dramatically reversed the Lys-induced decrease in GSH levels and rise in MDA levels. According to these findings, the Nrf2 signaling pathway is a crucial regulator of the Lys-induced ferroptosis that occurs in CRC cells. *In vivo* examination of Lys' antitumor capabilities revealed that injection of Lys significantly reduced tumor formation in mice harboring SW480 tumor xenografts, but Lip1 therapy lowered Lys' antitumor capability. The use of Lys and/or Lip1 had no impact on the mice's weight. In addition, histological evidence showed that treatments with Lys and/or lip1 did not result in toxicity in the main organs of mice. These organs include the lung, the heart, the liver, and the kidneys.

Here are the limitations of our study: First, we exclusively evaluated the impact of Lys on HCT116 and SW480 CRC cells using *in vitro* cell culture procedures, despite the fact that the human environment and *in vitro* conditions are quite different. Consequently, the findings of this investigation cannot alone inform therapeutic practice. We delivered just three doses of Lys, namely, 5, 15, and 30 mg, and observed only six, twelve, and 24 hours for three time points. This is incongruous with the biological rhythm and timing of therapy in CRC. We feel that repeated usage of Lys (4-6 h dosage interval) may one day be used as an adjuvant therapy for CRC. We cannot rule out the possibility that Lys is a predictor of intestinal damage in patients with colorectal cancer due to the fact that Lys experiments were conducted on the body of mice, whereas the rectal region is the primary site of CRC onset in humans, where the cell types and arrangement structure are quite different from mice.

**Figure 8:**
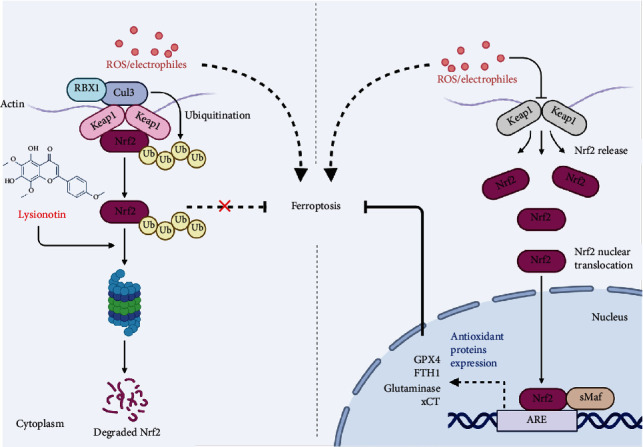
Schematic diagram of the pharmacology effects of Lys.

In summary, the findings of our study revealed that Lys inhibited the progression of colorectal cancer via induction of ferroptosis. We also provide evidence that Lys promotes the degradation of Nrf2 in proteosome, which contributes to its pharmacology effects in regulating ROS accumulation and ferroptosis (Figure 8), suggesting it can be used as a chemotherapeutic agent for the treatment of CRC.

## Figures and Tables

**Table 1:** The information of primers used in qPCR.

Gene	Forward primer (5′-3′)	Reverse primer (5′-3′)
Nrf2	CACATCCAGTCAGAAACCAGTGG	GGAATGTCTGCGCCAAAAGCTG
Keap1	CAACTTCGCTGAGCAGATTGGC	TGATGAGGGTCACCAGTTGGCA
GAPDH	GTCTCCTCTGACTTCAACAGCG	ACCACCCTGTTGCTGTAGCCAA

## Data Availability

The data used to support the findings of this study are included within the article.
